# Colorimetric detection of individual biothiols by tailor made reactions with silver nanoprisms

**DOI:** 10.1038/s41598-021-83433-4

**Published:** 2021-02-16

**Authors:** Pei Li, Sang Mo Lee, Hyo Yong Kim, Soohyun Kim, Steve Park, Ki Soo Park, Hyun Gyu Park

**Affiliations:** 1 Department of Chemical and Biomolecular Engineering (BK 21+ Program), KAIST, Daehak-ro 291, Yuseong-gu, Daejeon, 34141 Republic of Korea; 2 Department of Materials Science and Engineering, KAIST, Daehak-ro 291, Yuseong-gu, Daejeon, 34141 Republic of Korea; 3 Department of Biological Engineering, College of Engineering, Konkuk University, Seoul, 05029 Republic of Korea

**Keywords:** Diagnostic markers, Predictive markers, Prognostic markers, Imaging and sensing, Optical spectroscopy, Nanobiotechnology, Biosensors, Optical materials and structures

## Abstract

We herein described a rapid, sensitive, and selective colorimetric sensing platform for biothiols in human serum, which relies on the dual functions of biothiols as anti-etching and aggregating agent for silver nanoprisms (AgNPRs). In principle, the target biothiols that bind to the surface of AgNPRs through Ag–S covalent interactions protect the AgNPRs from being etched by chloride ion (Cl^−^) in human serum, thus exhibiting the blue/purple color that is indicative of AgNPRs. On the other hand, the color of AgNPRs turned to yellow in the absence of biothiols or the presence of non-sulfur-containing amino acids, indicating the formation of small silver nanoparticles (AgNPs). Importantly, we found that individual biothiols (Hcy, Cys, and GSH) exert not only the anti-etching effect, but also the aggregating effect on AgNPRs, which can be modulated by simply tuning the pH conditions, and this consequently allows for the discriminative detection of each biothiol. Based on this simple and cost-effective strategy, we successfully determined the Hcy, Cys, and GSH in human serum with high sensitivity and selectivity within 10 min, demonstrating the diagnostic capability and potential in practical applications.

## Introduction

The determination of sulfur-containing amino acids such as homocysteine (Hcy), cysteine (Cys), and glutathione (GSH) in human serum is important because the level of total biothiols can serve as the biomarker for predicting survival rates in oral cancer patients^1^, and monitoring oxidative stress in patients with nonalcoholic steatohepatitis^2^ and acute appendicitis^3,4^. Furthermore, the determination of individual biothiols is also required since each plays a vital role in the clinical diagnosis of many disorders and diseases^5^. For example, decreased Hcy levels in plasma are useful for the diagnosis of homocystinuria^6^, while the elevated Hcy levels in plasma can be a risk factor for osteoporosis^7^, Alzheimer’s disease^8^, occlusive vascular diseases^9,10^, cardiovascular diseases^11,12^, neural tube defects^13^, and lung cancer^14^. Moreover, Cys is recognized as a potential neurotoxin, and the deficiency of Cys is closely related with the depigmentation of hair, liver damage, skin lesions, edema, and the loss of muscle and fat^15–17^. Lastly, the deficiency of GSH is linked with the suppression of immune system functions^18^ and acceleration of aging^19^, and elevated GSH levels are associated with the increased resistance of cancer cells to chemotherapy^20^. Furthermore, an imbalanced level of GSH is implicated in neurodegenerative disorders, cystic fibrosis, and human immunodeficiency virus-related diseases. Therefore, the measurement of individual biothiols as well as total biothiols is quite important for early-stage monitoring of the health problems.

The gold standard methods for the determination of biothiols are high performance liquid chromatography (HPLC), capillary electrophoresis (CE), and mass spectrometry (MS), which measure the total biothiols while discriminating individual biothiols. However, HPLC and MS often require complicated procedures, high levels of technical expertise, and expensive instruments^21–25^. Even though CE is simple, inexpensive, and can be routinely performed by technicians, it requires relatively bulky instruments, limiting its application at point-of-care (POC) environments. In recent years, a number of simple, convenient, and cost-effective methods that are suitable for point-of-care-testing (POCT) have been devised for the determination of biothiols. The representative example is found in the colorimetric method that can detect biothiols even with the naked-eye^26^. Notably, silver nanoprisms (AgNPRs) are remarkably appealing due to their unique size and shape-dependent plasmonic/optical properties, which are different from the widely used nanoparticles such as silver and gold nanoparticles in previous colorimetric biosensing applications^27^. In addition, AgNPRs can be etched by heat^28^, hydrogen peroxide (H_2_O_2_)^29^, UV light^30^, and inorganic anions such as halide ions (I^−^, Br^−^ and Cl^−^), H_2_PO_4_^−^, and SCN^−^^31–35^, causing the morphology change accompanied by the blue-shift. Motivated by this phenomenon, various detection methods for biothiols that exert anti-etching effect on AgNPRs have been reported^36,37^. Although these methods are promising to detect the level of total biothiols, it is still challenging to distinguish individual biothiols in a simple, rapid, and cost-effective manner.

**Figure 1:**
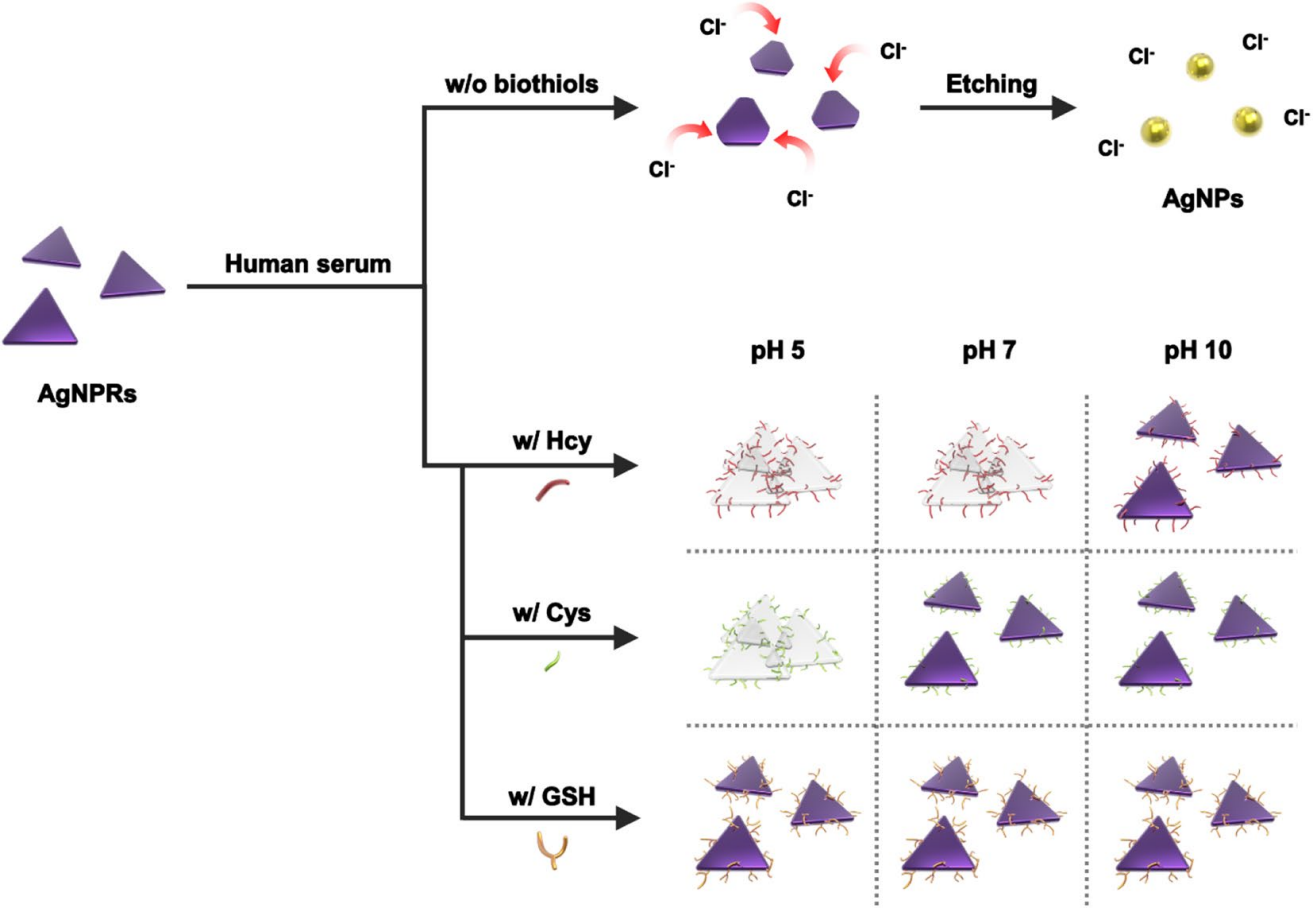
A schematic illustration of colorimetric detection system for individual biothiols based on the tailor made reactions with AgNPRs.

Herein, we propose a new method for the selective detection of both individual and total biothiols in human serum by utilizing AgNPRs. The overall detection procedure is illustrated in Fig. 1, which relies on the dual functions of biothiols as anti-etching and aggregating agent for AgNPRs. As shown in Fig. 1, at all pH conditions tested in this work (pH 5, 7, and 10), AgNPRs were etched by Cl^−^ in human serum when target biothiols are absent or non-sulfur-containing amino acids are present, which leads to the formation of small spherical silver nanoparticles (AgNPs) and, accordingly, its colorimetric signal is transitioned from blue/purple to yellow. However, the presence of biothiols that bind to the surface of AgNPRs through Ag–S covalent interaction prevents AgNPRs from being etched by Cl^-^ in human serum, thereby exhibiting the blue/purple color that is indicative of AgNPRs. Importantly, Hcy, Cys, and GSH can be discriminated from each other by simply tuning pH, which was not attempted in conventional AgNPR-based colorimetric assays^36,37^. Specifically, as compared to the anti-etching effect of biothiols that is consistent at all pH conditions, the aggregating effect of biothiols depends on the pH condition: at pH 5, both Hcy and Cys exhibited the aggregating effect, but at pH 7, only Hcy showed the aggregating effect, which are evidenced by the color transition from blue/purple to colorless. Moreover, all three biothiols (Hcy, Cys, and GSH) exerted the negligible aggregating effect at pH 10 (Table S1). By utilizing the tailor made reactions with AgNPRs based on these findings, we successfully determined the individual biothiols in human serum with great sensitivity and selectivity by simply measuring the colorimetric response.

## Results and discussion

### Feasibility of AgNPR-based biothiol detection

First, we verified the etching effect of Cl^-^ on AgNPRs by measuring the colorimetric response of AgNPRs at different concentrations of Cl^−^. The results in Fig. S1 show that the maximum absorption peak around 570 nm, which corresponds to the triangular shape of AgNPRs, completely disappears in the presence of Cl^-^ at concentrations over 500 µM. Instead, the new absorption peak around 416 nm that is indicative of small AgNPs appears, implying the shape transformation from AgNPRs to AgNPs. These results were also supported by the photographic images that show the colorimetric transition of AgNPRs from blue/purple to yellow. Based on these results, we assumed that the human serum that is known to contain *ca.* 100 mM Cl^−^ can be directly used as the etching agent for AgNPRs^39^.

**Figure 2:**
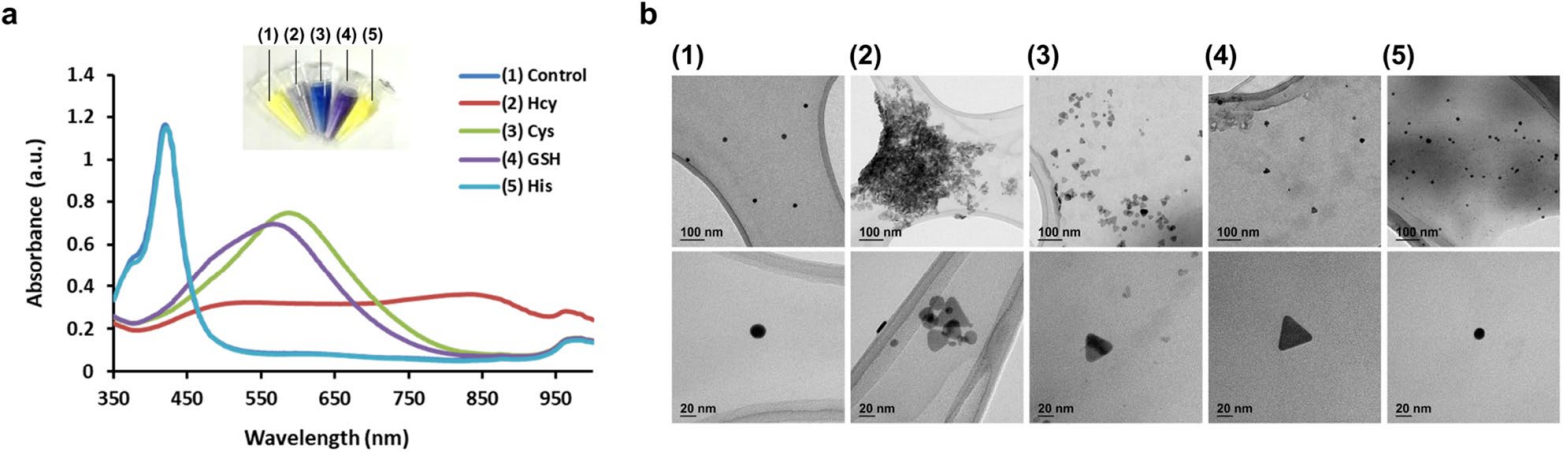
Feasibility of colorimetric detection of biothiols using AgNPRs in 1% human serum (pH 7). (**a**) Absorption spectra and corresponding photograph images and (**b**) TEM images of AgNPRs in 1% human serum (pH 7). (1) indicates the absence of target (control), and (2–5) indicates the presence of Hcy, Cys, GSH, and His, respectively. The final concentration of Hcy, Cys, GSH, and His was 6 µM.

Next, we validated the feasibility of this strategy by checking the effects of biothiols (Hcy, Cys, and GSH) and non-sulfur-containing amino acid (histidine, His) on the colorimetric signal of AgNPRs in human serum (pH 7). As expected, the human serum (1%) effectively exerted the etching effect on AgNPRs, which is evidenced by the dominant absorption peak around 416 nm. In contrast, the presence of Hcy, Cys, and GSH suppressed human serum-induced etching effect on AgNPRs, which were clearly distinguished from the sample with non-sulfur-containing amino acid (His) (Fig. 2a). Interestingly, Hcy caused the aggregation of AgNPRs and, accordingly, the maximum absorbance around 850 nm appeared, which is different from the other biothiols (Cys and GSH). The Hcy-induced aggregation is attributed to the intermolecular electrostatic interaction between Hcy molecules adsorbed on the surface of AgNPRs. Under the neutral pH condition (pH 7), Hcy enables the interparticle cross-linking through electrostatic interaction between COO^−^ and NH_3_^+^^40^, thus inducing the aggregation of AgNPRs. On the other hand, Cys that contains one less methylene group compared to Hcy binds tightly to the surface of AgNPRs, and hence the functional groups (COO^−^ and NH_3_^+^) of Cys are not fully exposed. As a result, the intermolecular electrostatic interaction between Cys molecules adsorbed on the surface of AgNPRs decreased, leading to the dispersion of AgNPRs. In the case of GSH, the interfacial reactivity of GSH is less favorable due to their relatively bulky molecular size and the steric hindrance from their glutamate moiety, and thus, GSH hardly exerts the aggregating effect on AgNPRs^41,42^. Overall, these results matched well with the one obtained by TEM analysis where the triangular shape of AgNPRs was observed in the presence of biothiols (2–4), but the aggregated form of AgNPRs was only observed in the presence of Hcy (2), confirming the anti-etching and aggregating effect of biothiols on AgNPRs (Fig. 2b).

### pH effect on AgNPR-based biothiol detection

**Figure 3:**
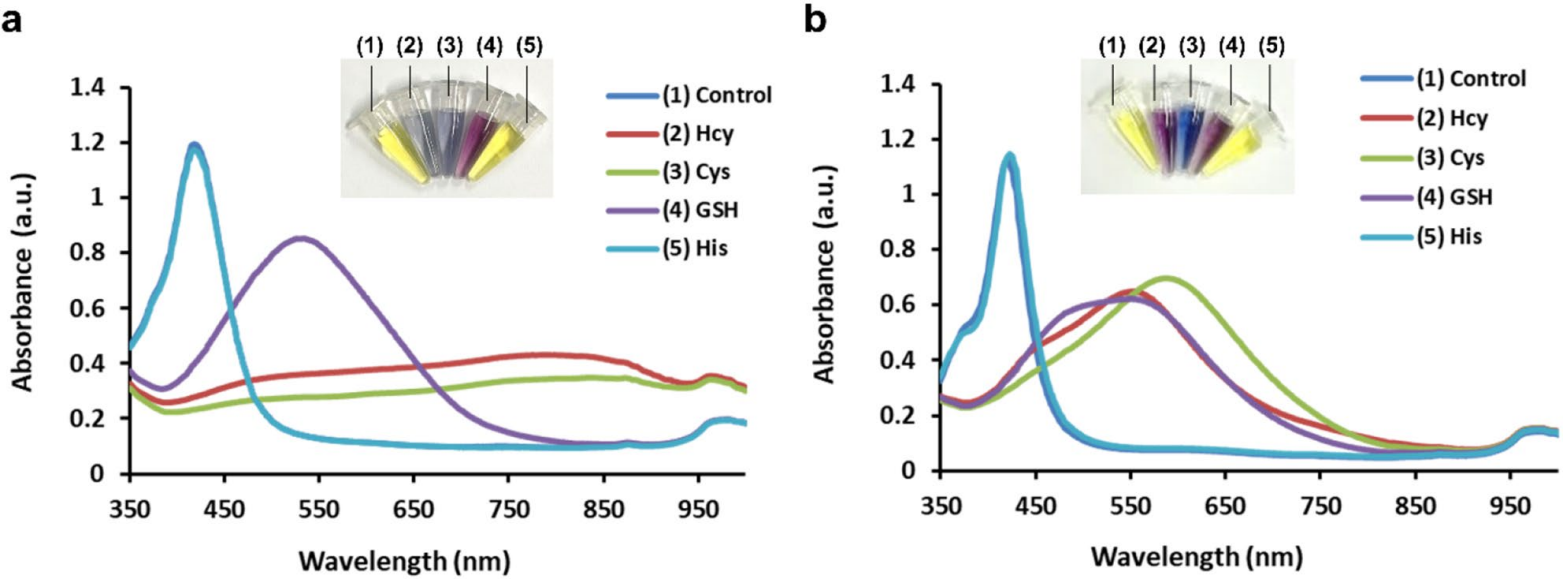
pH effect on AgNPR-based biothiol detection. Absorption spectra and corresponding photograph images of AgNPRs in 1% human serum at (**a**) pH 5 and (**b**) pH 10. (1) indicates the absence of target (control), and (2–5) indicates the presence of Hcy, Cys, GSH, and His, respectively. The final concentration of Hcy, Cys, GSH, and His was 6 µM.

We also investigated the pH effect on the detection of biothiols using AgNPRs in human serum. As shown in Fig. 3a, both Hcy and Cys induced the aggregation of AgNPRs in human serum (pH 5) and, accordingly, a new absorption peak around 850 nm appeared, indicating the formation of Hcy and Cys-mediated aggregates of AgNPRs, while AgNPRs were not aggregated in the presence of GSH. The aggregating effect of Cys at pH 5 is ascribed to its acidic pI (pI = 5) that enables Cys molecules to exist as zwitterions, enhancing the intermolecular electrostatic interaction between Cys molecules adsorbed on the surface of AgNPRs to induce the aggregation. However, the aggregating effect of Cys on AgNPRs is less effective than that of Hcy due to the structural difference from Hcy as explained above^43,44^. Moreover, all of the biothiols hardly induced the aggregation of AgNPRs in human serum at pH 10, because the intermolecular electrostatic interactions of biothiols are not favored due to the deprotonation of functional groups for all biothiols at the basic condition (Fig. 3b). These observations confirm that AgNPRs can be utilized as novel and simple probes for the analysis of biothiols. It should be noted that Hcy, Cys, and GSH exert the anti-etching effect on AgNPRs at all pH conditions, but their aggregating effects are dependent on the pH conditions. As summarized in Table S1, Hcy and Cys induced the aggregation of AgNPRs at pH 5, Hcy exclusively induced the aggregation of AgNPRs at pH 7, and no biothiols exerted the aggregating effect on AgNPRs at pH 10, which is ascribed to the difference in their molecular structures.

To demonstrate the feasibility of the proposed method for the discrimination of individual biothiols, we prepared the model samples containing different combinations of three biothiols (Hcy, Cys, and GSH) in human serum. As shown in Fig. S2a, at pH 7 where Hcy only exerts aggregating effect, the sample (red line) containing three biothiols (Hcy, Cys, and GSH) showed high absorbance at 850 nm, which is different from the one containing Cys and GSH (blue line), indicating the presence of Hcy. In addition, at pH 5 where Cys exerts aggregating effect, the sample containing three biothiols (Hcy, Cys, and GSH; red line) showed the increased absorbance at 850 nm, which is distinguished from the one containing Hcy and GSH (blue line; Fig. S2b), implying the existence of Cys. Finally, the absorbance at 416 nm was decreased in the presence of sample containing three biothiols (Hcy, Cys, and GSH; red line) compared to the one with Hcy and Cys, not GSH (Fig. S2c). As a result, not only the total biothiols but also individual biothiols can be discriminatively determined based on the colorimetric response of AgNPRs by simply tuning the pH condition, which has never been demonstrated.

### Selectivity test

**Figure 4:**
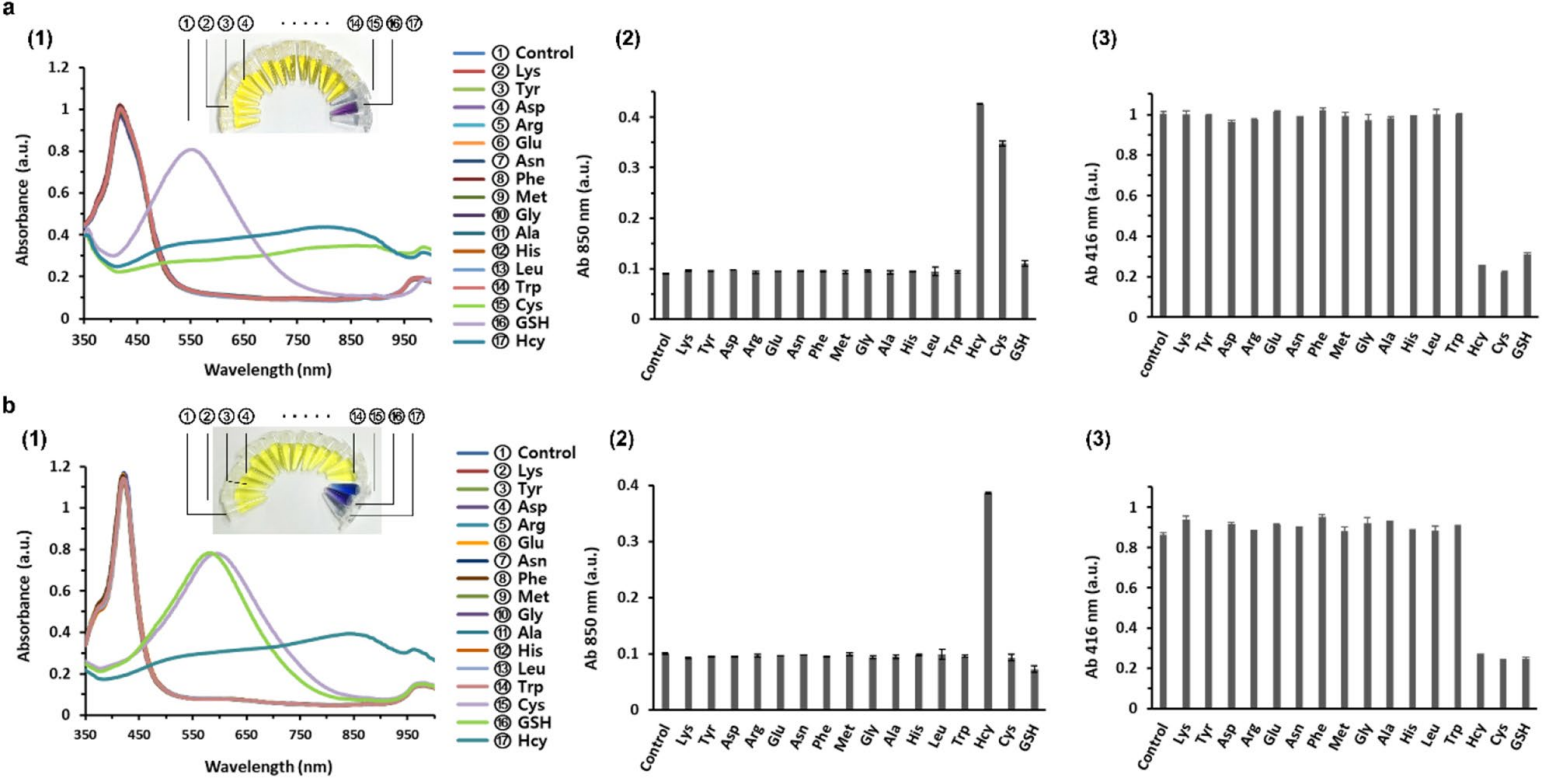
Selectivity of the AgNPR-based biothiol detection system. (1) Absorption spectra and corresponding photograph images, and absorbance at (2) 850 nm and (3) 416 nm of AgNPRs in the presence of non-sulfur-containing amino acids and biothiols in 1% human serum at (**a**) pH 5 and (**b**) pH 7, respectively. The concentration of non-sulfur-containing amino acids and biothiols was 9 µM.

To assess the specificity of the new detection strategy, 13 non-sulfur-containing amino acids and biothiols (Hcy, Cys and GSH) were tested at pH 5 and 7, and their aggregating and anti-etching effects were compared. As shown in Fig. 4, Hcy, Cys, and GSH can be successfully distinguished from non-sulfur-containing amino acids at both pH 5 and 7. In the presence of biothiols that exert the anti-etching effect, the color of AgNPRs was blue/purple, which is indicative of the triangular shape of AgNPRs. On the other hand, the color of AgNPRs in the presence of non-sulfur-containing amino acids turned from blue/purple to yellow, which indicates the formation of small AgNPs. As explained in the section ‘*pH effect on AgNPR-based biothiol detection*’, the new absorption peak at 850 nm, which is indicative of aggregated AgNPRs, appeared in the presence of Hcy and Cys at pH 5, while the peak at 850 nm appeared only in the presence of Hcy at pH 7. These results clearly demonstrate that this new sensing method is highly selective towards the target biothiols without any interference from other amino acids.

### Sensitivity test

**Figure 5:**
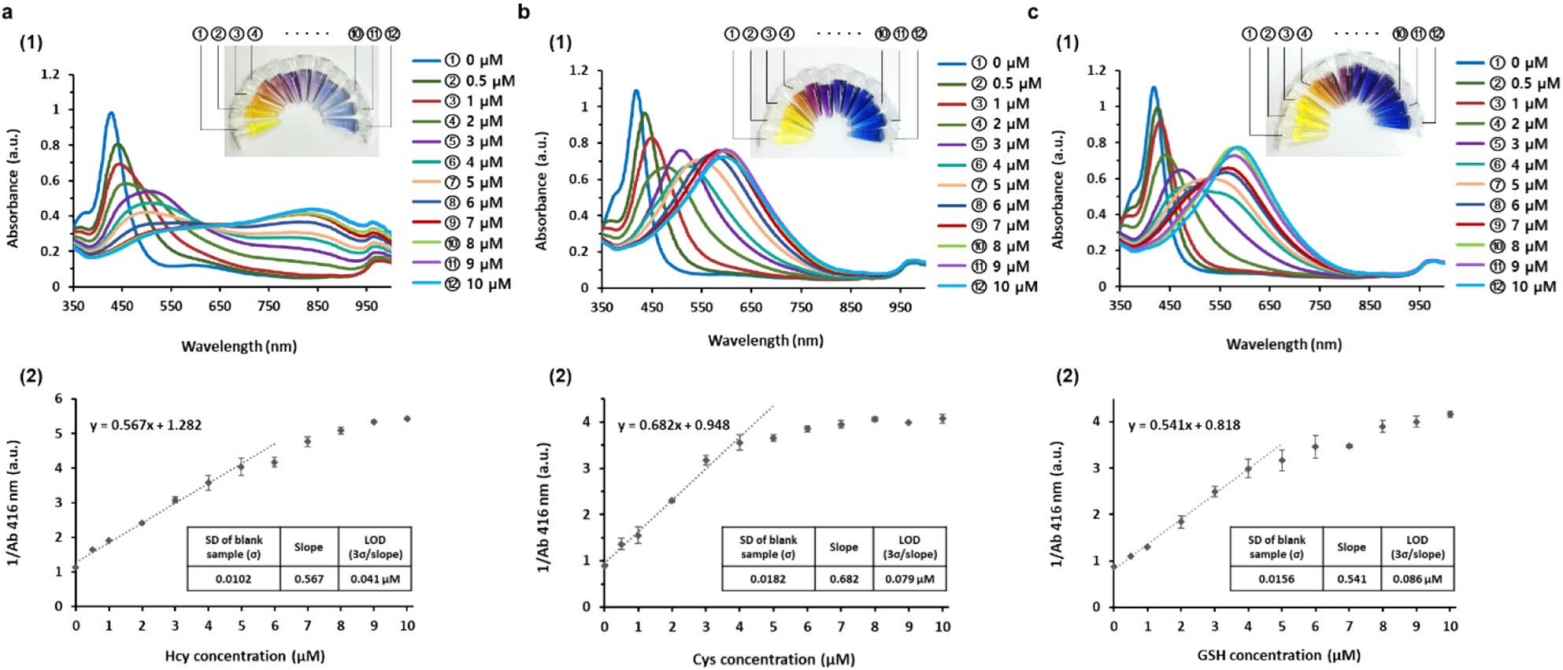
Sensitivity of AgNPR-based biothiol detection system. (1) Absorption spectra and corresponding photograph images and (2) the reciprocal absorbance at 416 nm in the presence of (**a**) Hcy, (**b**) Cys, and (**c**) GSH at varying concentrations (0–10 µM) in 1% human serum (pH 7), respectively. In (2), the calibration curves with the linear equation, and the tables showing standard deviation (SD) of the blank sample, slope of the linear calibration curve, and limit of detection (LOD) for (**a**) Hcy, (**b**) Cys, and (**c**) GSH, respectively, are inserted.

The detection sensitivity of the proposed system was determined by analyzing the absorption peak at 416 nm in human serum (pH 7), which is indicative of small AgNPs (Fig. 5a(1), Fig. 5b(1), Fig. 5c(1)). Specifically, the reciprocal absorbance at 416 nm as a function of target biothiol concentrations was selected for more accurate quantification of the target biothiols because it showed excellent linear relationship with the better R^2^ values compared with the absorbance at 416 nm or wavelength shifting. The results showed that the reciprocal absorbance at 416 nm proportionally increased with increasing concentrations of Hcy in the range of 0 to 5 µM (R^2^ = 0.99), but plateaued at concentrations higher than 5 µM (Fig. 5a(2)). The limit of detection (LOD) was 0.041 µM. In the same manner, Cys and GSH were determined down to 0.079 µM and 0.086 µM with the excellent linear relationship in the range of 0 to 4 µM (R^2^ = 0.99) and 0 to 4 µM (R^2^ = 0.996), respectively (Fig. 5b(2), Fig. 5c(2)). Notably, these LOD values are comparable to or even lower than those of other colorimetric methods to detect biothiols^44–46^.

### Recovery test

Finally, the proposed method was employed to measure the amount of total biothiols in human serum, and the recovery rate was calculated to further demonstrate the practical applicability of this system. First, the calibration curve was obtained by measuring the absorbance at 416 nm at different concentration of Cys in human serum, and the original concentration of biothiols in human serum was estimated by employing the standard addition method and using Cys as a standard (Fig. 5b(2))^47–49^. As shown in Table 1, the concentration of biothiols in the human serum was determined to be 144 µM, which is in good agreement with the previous reports^5,45^. Furthermore, we confirmed that this method is highly robust and reproducible as evidenced by the good recovery rate ranging from 94.4 to 108.7% and the low coefficient of variation (CV) less than 8%. These results prove the reliability of the proposed method for the determination of biothiols in the real and clinical samples.

**Table 1:** Determination of biothiols in 1% human serum.

Determined biothiols (µM)^a^	Added Cys (µM)	Measured Cys (µM)^b^	SD^c^	CV (%)^d^	Recovery (%)^e^
144	2	1.979	0.067	3.384	94.44
	3	3.261	0.178	5.445	108.69
	4	3.829	0.303	7.901	99.35

^a^The concentration of biothiols in human serum is determined by using the standard addition method (Fig. 5b). The concentration of Cys in human serum is measured to be 140.79 ± 9.42 µM using Cysteine Assay Kit (Fluorometric) (Abcam, United Kingdom), and the mean and standard deviation are estimated from triplicate measurements.

^b^Mean of three measurements.

^c^Standard deviation of three measurements.

^d^Coefficient of variation = SD/mean × 100.

^e^Measured value/added value × 100.

## Conclusions

We herein developed a simple and convenient strategy for the colorimetric detection of biothiols by utilizing AgNPRs as the key component. Through the systematic investigation, we found that the target biothiols protect AgNPRs from being etched by human serum at all pH conditions (pH 5, 7, and 10), but the biothiol-induced aggregation of AgNPRs is dependent on pH conditions. Specifically, only Hcy induces the aggregation of AgNPRs at pH 7, and both Hcy and Cys cause the aggregation of AgNPRs at pH 5, but nothing shows the aggregating effect on AgNPRs at pH 10. With the proposed colorimetric strategy, all biothiols can be discriminated from the non sulfur-containing biomolecules based on their anti-etching effect on AgNPRs, and even individual biothiols can be differentiated from each other by simply tuning the pH conditions. Importantly, the human serum that is the main biological source for the detection of biothiols can be directly analyzed without the need for additional etching agents because it contains sufficient concentration of Cl^−^. Furthermore, the proposed system does not require sophisticated detection instrumentation, complicated manipulations, and precise control of reaction temperature, which are suitable for the application in the facility-limited environment. Thus, we believe that the developed strategy provides a new perspective for rapid, direct, convenient, and cost-effective detection of sulfur-containing biomolecules in practical applications.

## Methods

### Materials

Silver nitrate (AgNO_3_) was purchased from Kojima Chemicals Co., Ltd. Polyvinylpyrrolidone powder (PVP, average M.W. ~ 29,000), sodium citrate (trisodium salt: dehydrate) (Na_3_CA, ≥ 99.5%), sodium borohydride (NaBH_4_, ≥ 98.0%), L-homocysteine (Hcy), L-cysteine (Cys), L-glutathione reduced (GSH), L-lysine (Lys), L-tyrosine (Tyr), L-glutamic acid (Glu), L-asparagine (Asn), L-phenylalanine (Phe), L-methionine (Met), glycine (Gly), L-alanine (Ala), L-histidine (His), L-leucine (Leu), and L-tryptophan (Trp), and human serum (from human male AB plasma) were purchased from Sigma-Aldrich. Hydrogen peroxide (H_2_O_2,_ 35%) was purchased from Junsei Chemical Co., Ltd. Centrifugal filters (Amicon Ultra-0.5 mL, Ultracel -3 K) were obtained from Millipore. All reagents were used without further purification. The ultrapure water was prepared from a Millipore Q-Gard water purification system (Milli-Q) with a filter membrane of 0.22 µm, which was used for the preparation of aqueous solutions of all the reagents.

### Synthesis of AgNPRs

Silver nanoprisms (AgNPRs) were synthesized according to the previous report with slight modification^38^. In brief, freshly prepared NaBH_4_ (100 mM, 0.7 mL) was added into an aqueous solution of AgNO_3_ (0.8 mM, 50 mL), Na_3_CA (30 mM, 3 mL), and PVP (0.7 mM, 3 mL). The resulting solution termed “silver seed solution” exhibits a yellow color, which was stirred overnight (more than 6 h) at room temperature. Next, freshly prepared NaBH_4_ (100 mM, 0.5 mL) and H_2_O_2_ solution (30%, 1.25 mL) were added into a flask containing 50 mL “silver seed solution” while stirring. A rapid color transition was observed from yellow to blue/purple within 1 min. The resulting solution contains AgNPRs, which were analyzed by field-emission transmission electron microscopy (TEM) (JEM-3010, JEOL) with an acceleration voltage of 300 kV. The samples for TEM analysis were prepared by dropping the solutions onto a carbon-coated copper TEM grid, followed by drying at room temperature.

### AgNPR-based biothiol detection

First, human serum was filtered with centrifugal filters (molecular weight cut-off; MWCO = 3 kDa) at 14,000 rpm for 10 min at room temperature. After the 50-fold dilution of filtered human serum with ultrapure water, 100 µL human serum (2%) was mixed with the reaction solution composed of 40 µL AgNPRs, 20 µL HEPES buffer (100 mM, pH 5, 7, and 10), and 40 µL target biothiol at varying concentrations. After the 10 min incubation at room temperature, UV–vis absorption spectra of the samples were recorded by using an Infinite 200 PRO microplate reader and TECAN i-control 1.7 (https://lifesciences.tecan.com/plate_readers/infinite_200_pro?p=Software, TECAN Group Ltd., Switzerland).

### Standard addition method

First, human serum was filtered with centrifugal filters (molecular weight cut-off; MWCO = 3 kDa) at 14,000 rpm for 10 min at room temperature. After the 50-fold dilution of filtered human serum with ultrapure water, 100 µL human serum (2%) was mixed with the reaction solution composed of 40 µL AgNPRs, and 20 µL HEPES buffer (100 mM, pH 5, 7, and 10). Next, 40 µL target biothiols at varying concentrations (a set of standard samples) was spiked into the reaction solution, and after the 10 min incubation at room temperature, UV–vis absorption spectra of the samples were recorded by using an Infinite 200 PRO microplate reader and TECAN i-control 1.7, which was used to draw the calibration curve (Fig. 5b(2)). Finally, the total biothiols in the filtered human serum and the added biothiols were calculated by using the obtained calibration curve (Table 1).

## Supplementary Information

Supplementary Information.
