# Supplementary material for: Colorimetric detection of individual biothiols by tailor made reactions with silver nanoprisms
Source: Sci Rep. 2021 Feb 16;11:3937. doi: 10.1038/s41598-021-83433-4 (PMC7886879; doi:10.1038/s41598-021-83433-4)
Supplement: Supplementary file 1 — Supplementary Information. [file 41598_2021_83433_MOESM1_ESM.pdf]

Electronic Supplementary Material for

Colorimetric detection of individual biothiols by tailor made reactions with silver nanoprisms

Pei Li,[a,b†] Sang Mo Lee,[a†] Hyo Yong Kim,[a] Soohyun Kim,[a] Steve Park,[b] Ki Soo Park,[c*] and Hyun Gyu Park[a*]

[a] Department of Chemical and Biomolecular Engineering (BK 21+ program), KAIST, Daehak-ro 291, Yuseong-gu, Daejeon 34141, Republic of Korea

[b] Department of Materials Science and Engineering, KAIST, Daehak-ro 291, Yuseong-gu, Daejeon 34141, Republic of Korea

[c] Department of Biological Engineering, College of Engineering, Konkuk University, Seoul 05029, Republic of Korea.

*To whom correspondence should be addressed.

E-mail: hgpark@kaist.ac.kr (H.G. Park); Phone: +82-42-350-3932; Fax: +82-42-350-3910.

E-mail: akdong486@konkuk.ac.kr (K.S. Park); Phone: +82-2-350-3742; Fax: +82-2-350-3742.

[†]These authors equally contributed to this work.

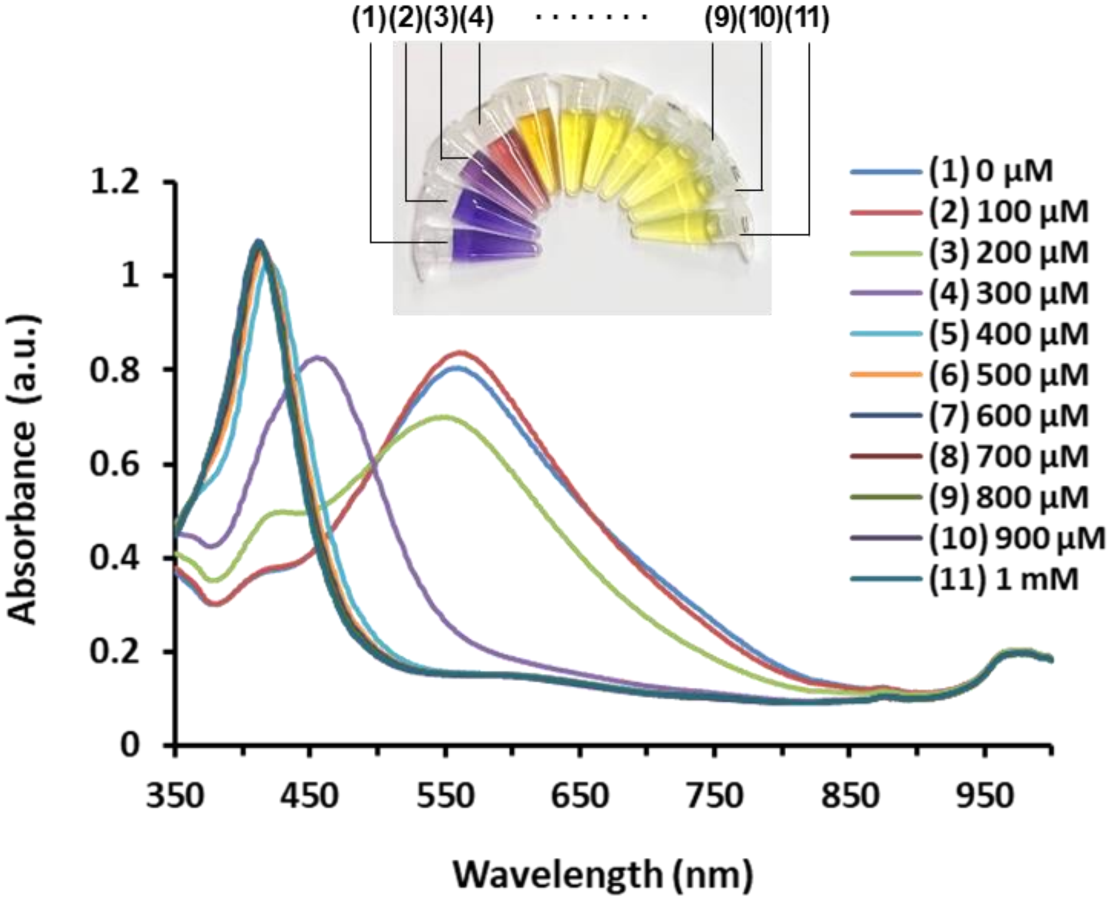

Fig. S1. Etching effect of Cl^- on AgNPRs in 10 mM HEPES buffer (pH 7). Inset: photograph images of AgNPRs incubated with NaCl (0-1 mM). The absorption peak around 570 nm indicates the existence of triangular AgNPRs [1].

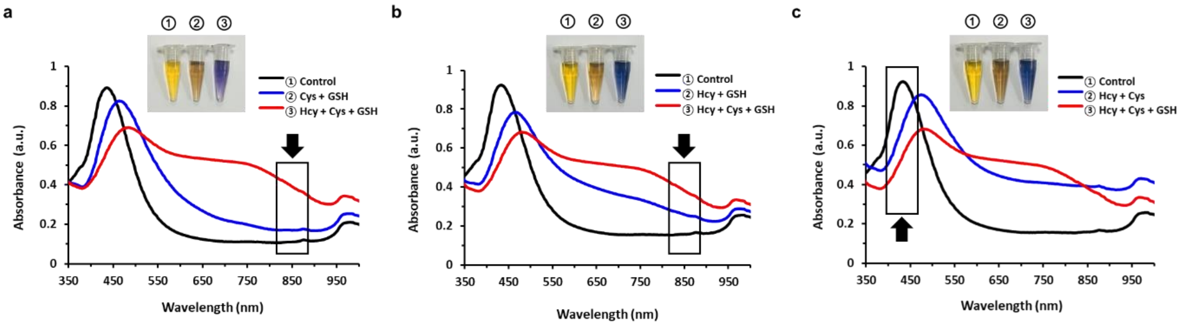

Fig. S2. Discrimination of individual biothiols using the AgNPR-based biothiol detection system. Absorption spectra and corresponding photograph images of AgNPRs in the presence of different combinations of biothiols in 1% human serum at (a) pH 7, (b) pH 5 and (c) pH 5, respectively. The concentration of each biothiol in the mixture was 2 μM.

Table S1. The aggregating effect of individual biothiols on AgNPRs at different pH conditions. At all pH conditions, the anti-etching effect of biothiols on AgNPRs was maintained.

	Hcy	Cys	GSH
pH 5	O	O	X
pH 7	O	X	X
pH 10	X	X	X

Table S2. Comparison of different analytical detection methods for Hcy, Cys, and GSH.

Key component	Signaling method	Target	Detection limit (μM)	Linear range (μM)	Limitation	Reference
Ce-MOF [a]	Colorimetry	Hcy, Cys, and GSH	0.143, 0.135, and 0.129	0-40	- Long preparation time<br>- No specificity to discriminate individual biothiols	[2]
PEI-AgNCs [b]	Fluorometry	Hcy, Cys, and GSH	0.047, 0.042, and 0.38	0.1-10, 0.1-10, and 0.5-6	- Complicated surface modification<br>- No specificity to discriminate individual biothiols	[3]
ACCA [c]	Fluorometry	Hcy, Cys, and GSH	0.09, 0.08, and 0.18	5-40, 5-40, and 0-40	- No specificity to discriminate individual biothiols	[4]
FSN-capped AuNPs [d]	Colorimetry	Hcy, Cys	0.2 and 0.8	0.5-2, and 1-4.5	- Relatively poor detection sensitivity<br>- No specificity to discriminate individual biothiols	[5]
Ascorbic acid-capped AuNPs	Colorimetry	Cys, GSH	0.0053 and 0.1	0.01-5, and 0.25-2.5	- No specificity to discriminate individual biothiols	[6]
AgNPRs	Colorimetry	Hcy, Cys, and GSH	0.041	0-5, 0-4, and 0-4		This work

[a] Cerium-metal-organic frameworks. [b] Polyethyleneimine-silver nanoclusters.

[c] 4-chloro-7-(diethylamino)-3-carbaldehyde coumarin. [d] Fluorosurfactant-capped gold nanoparticles

References

1. Aherne D., Ledwith D. M., Gara M. & Kelly J. M., Optical properties and growth aspects of silver nanoprisms produced by a highly reproducible and rapid synthesis at room temperature. Adv. Funct. Mater. 18 (14), 2005-2016 (2008).

2. Xiong Y. *et al.*, Synthesis of a mixed valence state Ce-MOF as an oxidase mimetic for the colorimetric detection of biothiols. Chem Commun. 51 (22), 4635-4638 (2015).

3. Zhang N., Qu F., Luo H. Q. & Li N. B., Sensitive and selective detection of biothiols based on target-induced agglomeration of silver nanoclusters. Biosens. Bioelectron. 42 (1), 214-218 (2013).

4. Dai X. *et al.*, A colorimetric, ratiometric and water-soluble fluorescent probe for simultaneously sensing glutathione and cysteine/homocysteine. Anal Chim Acta 900, 103-110 (2015).

5. Lu C. & Zu Y., Specific detection of cysteine and homocysteine: Recognizing one-methylene difference using fluorosurfactant-capped gold nanoparticles. Chem Commun (37), 3871-3873 (2007).

6. Bhamore J., Rawat K. A., Basu H., Singhal R. K. & Kailasa S. K., Influence of molecular assembly and NaCl concentration on gold nanoparticles for colorimetric detection of cysteine and glutathione. Sens. Actuators B Chem. 212, 526-535 (2015).
